# 

**Published:** 1847-10

**Authors:** 


### WESTERN JOURNAL

OF

### MEDICINE AND SURGERY.

### EDITORS.

DANIEL DRAKE, M. D.,

PROFESSOR OF PATHOLOGY AND THE PRACTICE OF MEDICINE IN THE UNIVERSITY OF LOUISVILLE.

LUNSFORD P. YANDELL, M. D.,

PROFESSOR OF CHEMISTRY AND PHARMACY IN THE SAME.

THOMAS W. COLESCOTT, M. D.

### BOOKS AND CORRESPONDENTS.

We have a number of books on hand of which we have not been able to prepare notices for the present number, and communications from valued correspondents which have been unavoidably crowded out. They will receive early attention.

### RECEIPTS OF MEDICAL JOURNAL FOR SEPT., 1847.

| | |
|---|---|
| Dr. F. Jones, Huntsville, Ala. | $5 00 |
| L. Brown Russellville, Tenn. | 5 00 |
| A. Gordon, Harrisburg, Mo. | 5 00 |
| W. L Gordon, Oak Grove, Mo. | 5 00 |
| S. Barbour, Rushville, Ind. | 25 00 |
| W. Long, New Maysville, Ind. | 5 00 |
| C. Mitchell, Clarksville, Ark. | 5 00 |
| J. Calhoun, Clark's Mills, Ark. | 5 00 |
| S. H. Dickson, Charleston, S. C. | 5 00 |

### COLLEGE OF PHYSICIANS AND SURGEONS

OF THE

The WESTERN JOURNAL OF MEDICINE and SURGERY is published monthly by the undersigned, at the corner of Main and Fifth streets, Louisville, at $5 per annum, payable in advance. Each number contains 96 pages, making two volumes in the year of more than 550 pages each.

Contributions to its pages by the Physicians of the Valley of the Mississippi, addressed to the Editors, are respectfully solicited.

Letters on business, to be addressed, postage paid, to the publishers.

*January*, 1844. PRENTICE & WEISSINGER.

TO OUR SUBSCRIBERS.—Another half year is gone, and a large sum is still due us for this Journal. We hope the *delinquent* subscribers will listen to the voice of conscience within them, and remit without delay.

Sept. 1847. PRENTICE & WEISSINGER.

